# Posttraumatic stress symptoms and health-related quality of life: a two year follow up study of injury treated at the emergency department

**DOI:** 10.1186/1471-244X-12-1

**Published:** 2012-01-09

**Authors:** Juanita A Haagsma, Suzanne Polinder, Miranda Olff, Hidde Toet, Gouke J Bonsel, Ed F van Beeck

**Affiliations:** 1 Department of Public Health, Erasmus Medical Center, Erasmus University Rotterdam, The Netherlands; 2 Center for Psychological Trauma, Department of Psychiatry, Academic Medical Center/de Meren, University of Amsterdam, The Netherlands; 3 Consumer Safety Institute, The Netherlands

**Keywords:** posttraumatic stress disorder, injury, functional outcome, quality of life

## Abstract

**Background:**

Among injury victims relatively high prevalence rates of posttraumatic stress disorder (PTSD) have been found. PTSD is associated with functional impairments and decreased health-related quality of life (HRQoL). Previous studies that addressed the latter were restricted to injuries at the higher end of the severity spectrum. This study examined the association between PTSD symptoms and health-related quality of life (HRQoL) in a comprehensive population of injury patients of all severity levels and external causes.

**Methods:**

We conducted a self-assessment survey which included items regarding demographics of the patient, accident type, sustained injuries, EuroQol health classification system (EQ-5D) and Health Utilities Index (HUI) to measure functional outcome and HRQoL, and the Impact of Event Scale (IES) to measure PTSD symptoms. An IES-score of 35 or higher was used as indication for the presence of PTSD. The survey was completed by 1,781 injury patients two years after they were treated at the Emergency Department (ED), followed by either hospital admission or direct discharge to the home environment.

**Results:**

Symptoms indicative of PTSD were associated with more problems on all EQ-5D and HUI3 domains of functional outcome and a considerable utility loss in both hospitalized (0.23-0.24) and non-hospitalized (0.32-0.33) patients. Differences in reported problems between patients with IES scores higher or lower than 35 were largest for EQ-5D health domains pain/discomfort (82% versus 28%) and anxiety/depression (53% versus 11%) and HUI domains emotion (92% versus 33%) and pain (84% versus 38%). After adjusting for potential confounders, PTSD remained strongly associated with adverse HRQoL.

**Conclusions:**

Among patients treated at an ED posttraumatic stress symptoms indicative of PTSD were associated with a considerable decrease in HRQoL in both hospitalized and non-hospitalized patients. PTSD symptoms may therefore raise a major barrier for full recovery of injury patients of even minor levels of severity.

## Background

Posttraumatic stress disorder (PTSD) may result from any event that involves an injury, or threatened or actual death. Regarding injury victims PTSD prevalence rates up to 37% have been found three months after the injury [1]. At long-term follow-up (> 1 year) PTSD prevalence rates from 5% [2] to 32% [3] have been reported.

A substantial share of studies that investigated prevalence rates and predictors of PTSD following injury addressed certain injury subgroups, such as victims of motor vehicle accidents [4-7], burn victims [8-10] or patients who required admission to hospital or the Intensive Care Unit [3,11-15]. Those previous studies were mainly conducted in clinical patient populations and were therefore restricted to accidents and injuries at the higher end of the severity spectrum.

PTSD generally originates from cumulative exposure to traumatic stressors, which also influence the probability of spontaneous remission from PTSD [16,17]. The level of traumatic stressors in the population of study may therefore affect to a large extent the prevalence rates found in studies on injury victims and which focus on a single stressor.

PTSD is associated with functional impairments and decreased health-related quality of life (HRQoL) [18,19]. In one of the scarce studies addressing the latter, Holbrook et al. [20] showed that in a subgroup of injury patients admitted to a trauma centre PTSD has a substantial impact on health-related quality of life. Similar results were found among adolescents and children [21,22]. However, these studies were again restricted to victims at the higher end of the severity spectrum and the association between PTSD and health-related quality of life among a comprehensive population of injury patients has yet to be studied.

The objective of this study was to assess the association between posttraumatic stress symptoms indicative of PTSD and HRQoL among this comprehensive injury population.

## Methods

### Study design

A patient-follow-up study, which was previously published [23], was conducted among a population-based sample of injury patients of all severity levels. This study followed injury patients aged 15 years and older who attended the ED of the Dutch Injury Surveillance System (a representative continuous registry of intentional and unintentional injuries of 17 hospitals in the Netherlands). Surveys were conducted at 2 months, 5 months, 9 months and two years after initial treatment. This study was conducted with the approval of the Ethics Committee Erasmus MC University Hospital.

### Subjects

Between 8 October 2001 and 31 December 2002 a sample was selected of 8,564 patients aged 15 years and older who attended the ED of the Dutch Injury Surveillance System [23]. The patients were treated at the ED, followed by either hospital admission or direct discharge to the home environment. The sample of patients consisted of victims of traffic, home and leisure, occupational and sport accidents. The sustained injuries varied from minor to severe injury, single and multiple injury and hospitalized and non-hospitalized patients. The sample of patients was stratified, over sampling patients who were hospitalized. Each injury patient of the selected sample received a postal questionnaire 21/2 months after the injury and 3,167 (37%) responded. The first questionnaire was made anonymous for privacy reasons. At 5, 9 and 24 months a follow-up questionnaire was sent to patients that responded to the preceding questionnaire. For these questionnaires the patients needed to give permissions by an informed consent form. The 5, 9 and 24 months follow-up questionnaire were completed by respectively 2,384, 2,295 and 1,781 patients. The present study used a sample of 1,781 respondents (i.e 21% of the original sample) on the two year post-trauma survey, which assessed both posttraumatic stress symptoms and HRQoL [23]. To adjust the data for non-response, a non-response analysis was conducted [23]. Multivariate logistic regression analysis was used to examine if variables age, sex, type of injury, external cause of the injury, hospitalization and length of stay, health status and ambulance transport were possible determinants of non-response. The significant variables were used to adjust for response bias by inverse probability weighting [24]. Additionally, the data were adjusted for stratification of the sample of ED patients [23]

### Questionnaire

The follow-up questionnaire included items regarding demographics of the patient, accident category, type of injury, health care use and the Impact of Event Scale (IES), which was used to assess symptoms of posttraumatic stress indicative of PSTD [25]. The IES consists of 15 items, which measure intrusive re-experiences of the trauma and avoidance of trauma-related stimuli. By combining the 15 items the total IES-score, ranging from 0 through 75, can be calculated. Wohlfarth et al. showed that a cut-off score of 35 on the total IES-score produced a sensitivity of .89, and a specificity of .94 against the DSM-IV diagnostic criteria for PTSD as the gold standard [26]. Therefore, we assumed that an IES-score higher than 35 (IES ≥ 35) represents symptoms of posttraumatic stress indicative of PTSD. The Dutch translation of the IES has been found to be valid and reliable [27].

Additionally, the questionnaire included items to measure functional outcome and HRQoL. HRQoL is an index of perceived functional outcome of an illness and disability that is anchored between 0 (worst imaginable health state or death) and 1 (full health), thus allowing comparison between the health status of patients with distinct diseases. To measure HRQoL, multi-attribute utility instruments (MAUIs) such as the Health Utility Index (HUI) or the EQ-5D may be used [28,29]. These instruments require the patient to report his or her health state with a standardised generic health state classification system, which is then converted into a health utility score using utility weights derived from the general population. Despite the similarities in obtaining the health utility score, there are important variations between the instruments regarding the health domains included in the health classification system and the methods applied to derive the utility weights [30]. As a result of these variations, the distinct instruments yield different utilities for similar health states. To overcome omissions in measuring HRQoL it is important to use several instruments that have complementary health domains [31].

Therefore, to measure functional outcome and HRQoL, the questionnaire included the EQ-5D and the HUI mark 3 (HUI3). With the EQ-5D classification system, respondents describe their health in three levels of severity on the health domains mobility, self-care, usual activities, pain/discomfort and anxiety/depression [32]. Subsequently, the weight of that health state is computed by a formula that firstly yields a partial weight score for each domain depending on the reported level and secondly adds the utility weights (also referred to as the 'tariff'), which are based on preference data of the general population of the UK [33].

For instance, a patient reports some problems with walking and performing usual activities, as well as moderate pain or discomfort (EQ-5D profile 21221). Full health has a utility value of 1. Because the health state of the patient deviates from the best possible health state (EQ-5D profile 11111), a fixed reduction of -.081 is applied. For the problems with walking, performing usual activities and moderate pain or discomfort reductions are applied of -0.069, -0.036 and -0.123 respectively. This is results in a utility of 0.691. The complete algorithm to calculate EQ-5D utilities is published by Dolan et al. [34]

The questionnaire included 19 items regarding the presence of one or more chronic disease(s) prior to the injury to assess comorbidity [35]. Comorbidity is defined as the presence of any coexisting medical conditions or disease processes additional to the injury that the injury patients sustained [36].

### Primary data-analysis

For analysis of the data the Statistical Package for the Social Sciences version 14.0 was used (SPSS Inc, Chigaco, Ill). The IES-score can be calculated if all IES items are completed. In 8% of the cases data of one of the 15 IES items was missing. For these cases, the missing IES item was estimated by calculating the median value of 5 nearby points. The missing data was then imputed by the estimated values [37]. If more than one of the 15 IES items was missing, data were not imputed. Chi-square statistics (dichotomous variables) and Student t tests (continuous variables) were used to test for differences between injury patients with IES scores higher or lower than 35.

Univariate logistic regression and multivariate logistic regression analyses (enter method) were used to determine the predictive value of patient demographics, accident category and severity level of the sustained injuries with regard to posttraumatic stress symptoms indicative of PTSD (IES ≥ 35) at two-year post-trauma. To dichotomize severity level, the injury diagnoses were categorized into two severity classes (mild versus moderate to severe) as previously tested by an international expert group [38]. The injury severity class moderate to severe comprises injuries such a skull-brain injury, fracture/dislocation of the vertebral column, fracture of pelvis and hip fracture. The injury severity class mild comprises injuries such as superficial injury, concussion and wrist fracture.

For the analysis of the association between IES ≥ 35 and HRQoL, we selected participants that filled in both EQ-5D and HUI3. To test differences between participants with and without PTSD regarding their responses on each of the EQ-5D and HUI3 health domains, the non-parametric Wilcoxon-Mann-Whitney test was conducted. Differences regarding the mean EQ-5D and HUI3 summary scores were tested with a one-way ANOVA. P-values < 0.05 were considered to indicate statistical significance.

Stepwise multiple regression analyses (enter method) was applied to investigate the association between demographics (block 1), hospitalization and comorbidity (block 2) posttraumatic stress symptoms indicative of PTSD (IES ≥ 35) (block 3) and HRQoL measured with the EQ-5D and HUI3.

## Results

### Study population

Regarding the respondents on the 24-month follow-up questionnaire, the average age was 44.5 years old and 46% were female. Over one half (54%) was injured due to home and leisure accidents. The sustained injuries of all the respondents consisted mostly of superficial injury/open wounds (51%) and upper extremity fractures (13%). After treatment at the ED, 9% of the respondents were admitted to hospital. Approximately one third (31%) had one or more pre-existing comorbid conditions. Table 1 shows the characteristics of the injury patients, accident category and hospitalization status.

**Table 1:** Characteristics of the injury patients, accident category and hospitalization status

Characteristics	(n = 1781)*^a^*
Patient demographics	
Age	44.9 (sd*^b ^*23.1)
Female sex	46%
Comorbid disease	31%
	
Accident category	
Home and leisure	54%
Traffic	16%
Occupational	13%
Sport	16%0
	
Hospitalization	8%

^a Weighted for stratification of the sample of injury patients and non-response.^

^b sd = standard deviation^

### Association of posttraumatic stress symptoms indicative of PTSD (IES ≥ 35) with HRQoL

With reference to the 1,781 respondents that completed the 24-month follow-up questionnaire, 1,585 (89%) filled in the EQ-5D and the HUI3 and 1,380 (77.5%) filled in the IES.

EQ-5D - Table 2 shows the responses on the EQ-5D of injury patients with IES scores higher or lower than 35. The calculated mean EQ-5D summary score for injury patients with IES scores ≥ 35 was 0.56, whereas for injury patients with lower IES scores the mean EQ-5D summary score was 0.87 (t = 112.0; p < 0.001). Respondents with posttraumatic stress symptoms indicative of PTSD reported significantly more problems on all five EQ-5D health domains (p < 0.001). Differences in reported problems between patients with IES scores higher or lower than 35 were largest for EQ-5D health domains pain/discomfort (82% versus 28%) and anxiety/depression (53% versus 11%).

**Table 2:** Mean utility scores and percentage of reported problems on the EQ-5D and HUI3 health domains of the respondents without and with posttraumatic stress symptoms (PTSS) indicative of posttraumatic stress disorder

	No posttraumatic stress symptoms (IES-score < 35; n = 1708)	Posttraumatic stress symptoms (IES-score ≥ 35 n = 73)	p
**EQ-5D**			
Mean EQ-5D utility score	0.87 (sd^a ^0.15)	0.56 (sd^a ^0.26)	< 0.001
Problems with mobility	15.4%	47.0%	< 0.001
Problems with self-care	5.1%	18.9%	< 0.001
Problems with usual activities	16.9%	53.2%	< 0.001
Pain/discomfort	28.1%	82.3%	< 0.001
Anxiety/depression	11.4%	53.9%	< 0.001
**HUI3**			
Mean HUI3 utility score	0.83 (sd^a ^0.24)	0.51 (sd^a ^0.26)	< 0.001
Problems with vision	54.1%	65.2%	< 0.001
Problems with hearing	8.6%	1.6%	< 0.001
Problems with speech	4.6%	28.7%	< 0.001
Problems with ambulation	12.8%	20.4%	< 0.001
Problems with dexterity	10.6%	23.0%	< 0.001
Problems with emotion	32.5%	91.6%	< 0.001
Problems with cognition	14.3%	52.6%	< 0.001
Pain	38.4%	84.4%	< 0.001

^a sd = standard deviation^

When the responses of hospitalized and non-hospitalized injury patients with IES ≥ 35 and IES < 35 are presented separately, again patients with symptoms indicative of PTSD (IES ≥ 35) report significantly more problems on each of the EQ-5D health domains, resulting in a mean EQ-5D utility loss of 0.32 for non-hospitalized patients (t = 112.2; p < 0.001) and 0.23 for hospitalized patients (t = 22.1; p < 0.001). Compared to the injury patients without PTSD indications, injury patients with symptoms indicative of PTSD (IES ≥ 35) at 24-months post-trauma also had significantly lower mean EQ-5D utility scores at the 21/2-month (t = 105.0, p < 0.001), 5-month (t = 100.1, p < 0.001) and 9-month (t = 38.1, p < 0.001) follow-up.

**Figure 1:**
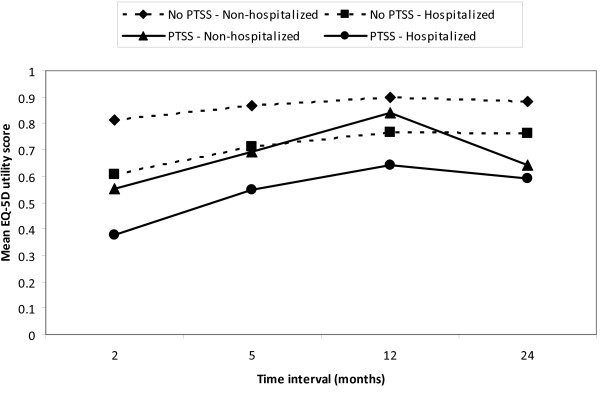
**Mean EQ-5D utility score of non-hospitalized and hospitalized patients**. with and without symptoms indicative of post-traumatic stress disorder (PTSS) at 21/2, 5, 12 and 24 month follow-up.

Figure 1 shows the mean EQ-5D utility score of non-hospitalized and hospitalized patients with and without symptoms indicative of PTSD at 21/2, 5, 12 and 24 month follow-up

HUI3 - Table 2 also shows the responses on the HUI3 domains reported by injury patients with IES-scores higher or lower than 35. For patients with IES ≥ 35 the calculated mean HUI3 summary score was 0.51 and for patients with lower IES-scores 0.83 (t = 81.1; p < 0.001). Respondents with posttraumatic stress symptoms indicative of PTSD (IES ≥ 35) reported significantly more problems on all HUI3 health domains, except hearing where a reverse association was found (p < 0.001). Differences in reported problems between patients with IES ≥ 35 and IES < 35 were largest for the HUI3 health domains emotion (92% versus 33%) and pain (84% versus 38%). Analysing the responses of non-hospitalized and hospitalized patients with and without PTSD indications (IES ≥ 35) separately shows that hospitalized patients with symptoms indicative of PTSD (IES ≥ 35) reported most problems. Non-hospitalized patients with lower IES-scores reported least problems on the HUI3 health domains. Symptoms indicative of PTSD (IES ≥ 35) were associated with a mean utility loss of 0.33 in non-hospitalized patients (t = 80.8; t < 0.001) and 0.24 in hospitalized patients (t = 15.9; t = 0.001)

The models tested to predict HRQoL measured with EQ-5D and HUI3 were both statistically significant (EQ-5D: F = 80.27, p < 0.001; HUI3: F = 118.55, p < 0.001). Table 3 shows that posttraumatic stress symptoms indicative of PTSD (IES ≥ 35) are associated with decreased HRQoL, even after controlling for possible confounders.

**Table 3:** Predictors of health-related quality of life at 2-year follow-up^§^

	Predictors	R2	Standardized B	p
**EQ-5D**	Age		-0.023	0.335
	Sex		-0.134	< 0.001
	Hospitalization		-0.246	< 0.001
	Comorbidity		-0.238	< 0.001
	PTSS		-0.234	< 0.001
		0.217		
**HUI3**	Age		-0.159	< 0.001
	Sex		-0.058	0.008
	Hospitalization		-0.098	< 0.001
	Comorbidity		-0.371	< 0.001
	PTSS		-0.211	< 0.001
		0.278		

**§ **Analysis based on stepwise multivariate regression analysis with demographics (age, sex) as block 1; comorbidity, hospitalization and severity level of the injury as step 2, and posttraumatic stress symptoms (PTSS) indicative of posttraumatic stress disorder as step 3.

## Discussion

Posttraumatic stress symptoms indicative of PTSD were associated with more problems on almost all domains of functional outcome and a considerable decrease of HRQoL in both non-hospitalized and hospitalized injury patients two years post-injury.

Previous studies on PTSD and HRQoL were conducted in clinical patient populations and were therefore restricted to accidents and injuries at the higher end of the severity spectrum [20-22]. This study was not restricted to particular injury subgroups, such as adolescent victims or victims with severe injuries [4,8,11,12,20]. The high variety in injuries included in this study and the relatively large sample size allowed examination of the association of a number of injury characteristics and posttraumatic stress symptoms indicative of PTSD.

We found that injury patients with posttraumatic stress symptoms indicative of PTSD reported significantly more problems on all EQ-5D and almost all HUI3 health domains. A study that investigated HRQoL with EQ-5D among patients with PTSD following cardiac arrest reported similar findings [39]. Among adolescent victims PTSD was associated with impairments in Role/Social Behavioral, Role/Social Physical, Bodily Pain, General Behavior, Mental Health, and General Health Perceptions subscales of the 87-item Child Health Questionnaire [22]. The resulting EQ-5D and HUI3 utility scores of injury patients with PTSD found in the current study are approximately in the range of the utility scores that Holbrook et al. derived with the multi-attribute utility instrument Quality of Well-being scale (QWB) (0.58 - 0.62) [20]. Although the HUI3 instrument yielded significantly lower health utility scores compared to the EQ-5D, which accords with results of other studies [40-42], both HUI3 and EQ-5D showed that PTSD was associated with a mean utility loss of 0.17 - 0.25. This concurs with the utility loss of anxiety disorders social phobia, generalized anxiety disorder and agoraphobia [43].

It should be noted that Holbrook et al. focused on injury patients admitted to a trauma centre with a length of stay of more than 24 hours and patients injured due to unintentional and intentional injury, whereas the current study included all admitted injury patients to general and university hospitals who were injured due to unintentional injury. Moreover, Holbrook et al. used an IES-score greater than 24 to identify patients with PTSD, whereas in the current study a cut off of 35 was used. Evidence from studies on this matter suggests that to avoid overestimation of the number of cases with PTSD, an IES-score of greater than 35 is more appropriate [26,44]. Using the DSM-IV as the diagnostic criteria for PTSD, a cut-off score of 35 produced sensitivity of .89, specificity of .94 [26]. With a cut-off point of 24, the sensitivity is 0.91 and the specificity 0.46 [45]. To avoid over diagnosing of PTSD in a comprehensive population with a relative low PTSD prevalence, it is important to use a high IES cut-off score that incurs a high specificity.

Nonetheless, an important shortcoming of this study was that existence of PTSD symptoms was measured with the IES rather than Clinician-Administered PTSD Scale for DSM-IV (CAPS). The IES is a self-report questionnaire that measures only two of the three main PTSD symptoms, namely intrusion and avoidance. It is not a diagnostic tool, i.e., it is not designed to diagnose mental disorders according to the DSM-IV (the fourth edition of the diagnostic and statistical manual for psychiatric disorders). Consequently, cases that in the current study were identified as having PTSD symptoms might not meet the DSM-IV criteria of clinical PTSD, and inversely. Due to differences in assessment of PTSD symptoms it is difficult to compare the results found in this study to previous studies on PTSD and health-related quality of life (HRQoL).

Both hospitalized and non-hospitalized injury patients with symptoms indicative of PTSD at 24 months post-injury reported a decrease in health status after 9 months, which may indicate that the sample is starting a deterioration process. On the other hand, patients might have overestimated their 9-month health status (and possibly also their 5-month health status), because their frame of reference has changed as a result of a temporary decrease in health status after the injury (response shift) [46,47]. However, without information on PTSD status at previous measure points, the reasons for the reductions in HRQoL at 24-months post-injury can only be speculated about.

Functional consequences of injury, both temporary and permanent, show large variations dependent on the injury location and injury type. In the current study we used the European injury classification EUROCOST [48]. This classification is compatible with the International Statistical Classification of Diseases, Injuries and Causes of Death - Ninth revision (ICD-9) classification system an consists of 39 injury groups that are homogeneous in terms of healthcare use, disability, as well as treatment and prognosis. In terms of anatomical classification the EUROCOST classification is simple compared to the ICD, which provides very detailed information on injury diagnoses by location and type of injury.

A second limitation of this study was the low response rate of the follow-up questionnaires [23]. The 24-month follow-up questionnaire, which included the IES, was send only to those patients who responded to the preceding three follow-up questionnaires send at 21/2, 5 and 9 months. This meant that only 21% of the patients of the initial sample selected for the follow-up study filled in the 24-month follow-up questionnaire. However, the data were adjusted for non-response and possible response bias, because the PTSD prevalence rates were calculated using data that were weighted with respect to the original sample size and composition by inverse probability weighting. For some aspects, such as the severity of sustained injuries, the adjustments of non-response could be improved, since injury severity scores were not available.

Evidence suggested that patients with very severe health problems are less likely to respond to a survey [49]. Differential underreporting by level of severity cannot be excluded, since we found a larger proportion of hospitalized patients among those with PTSD at 2 years post-injury. This could partly be caused by missing a larger share of the more severely injured hospitalized patients among those without PTSD (e.g. comatose patients). This may have led to a slight overestimation of the utility losses due to PTSD. However, severely injured patients are only a minor part of the total sample and PTSD remained significantly associated to adverse HRQoL, even after adjustment for confounders including hospitalization status.

In the current study PTSD is measured at 24 months follow-up only. A longitudinal study on PTSD and HRQoL among injury patients might elucidate any causal relationship between PTSD and subsequent reduced HRQoL. Furthermore, the influence of earlier HRQoL on PTSD remains to be investigated.

## Conclusions

We conclude that among patients admitted to an ED due to injuries of all causes and severity levels posttraumatic stress symptoms indicative of PTSD are associated with decreased HRQoL even after correction for possible confounders such as comorbidity. PTSD seems a major barrier for full recovery of injury patients of even minor levels of severity, and the development and evaluation of ED based policies for its early diagnosis and treatment should therefore be stimulated.

## Abbreviations

ED: emergency department; HRQoL: health-related quality of life; HUI: health utilities index; IES: impact of event scale; MAUI: multi-attribute utility instrument; PTSD: posttraumatic stress disorder.

## Conflict of Interest

The authors declare that they have no competing interests.

## Authors' contribution information

JAH executed the statistical analysis and drafted the manuscript. SP participated in the design of study, assisted with the statistical analysis and drafting of the manuscript. HT participated in the design of the study and data collection. MO participated in the design of study and drafting of the manuscript. GJB participated in the design of study and drafting of the manuscript. EFvB supervised, participated in the design of study and drafting of the manuscript. All authors read and approved the final manuscript.

## Funding

No financial support was received for the work.

## Pre-publication history

The pre-publication history for this paper can be accessed here:

http://www.biomedcentral.com/1471-244X/12/1/prepub
